# Fabrication and catalytic properties of ordered cyclopalladated diimine monolayer : investigation on catalytic mechanism†

**DOI:** 10.1039/c8ra06365f

**Published:** 2018-09-12

**Authors:** Linhong Wang, Pingping Huang, Jun Yang, Tiesheng Li, Luyuan Mao, Minghua Liu, Yangjie Wu

**Affiliations:** College of Chemistry and Molecular Engineering, The Key Lab of Chemical Biology and Organic Chemistry of Henan Province, The Key Lab of Nano-information Materials of Zhengzhou Zhengzhou 450001 P. R. China lts34@zzu.edu.cn +86-371-67766667; College of Materials Science and Engineering, Zhengzhou University Zhengzhou 450001 P. R. China; Beijing National Laboratory for Molecular Science, Institute of Chemistry, Chinese Academy of Sciences Beijing 100190 P. R. China

## Abstract

“Channel-like” self-assembled monolayers having aliphatic and aromatic diimines (denoted as Si@1DIS, Si@2DIS and Si@3DIS) immobilized on substrates and their palladacycle monolayers (Si@1DIS-Pd, Si@2DIS-Pd and Si@3DIS-Pd) were prepared and characterized. Their catalytic performances were investigated using the Suzuki coupling reaction as a model. Si@3DIS-Pd showed the highest catalytic activity in water without ligands, and better recyclability than that of Si@2DIS-Pd and Si@1DIS-Pd. The reason was the carbon in the aliphatic diimine of Si@2DIS-Pd and Si@1DIS-Pd was easily hydrolyzed because of the active hydrogen of α-C, resulting in poor recyclability. Control of the amount of catalyst could be achieved by modulating the diameter of the channel-like structure, which also affected the catalytic activity. The catalytic process and mechanism were investigated systematically and proposed based on the experimental results obtained by the water contact angle, ultraviolet spectroscopy, X-ray photoelectron spectroscopy, cyclic voltammetry and atomic force spectroscopy. Changes in the morphology of monolayer surfaces during the catalytic process with or without stirring presented a clear process from order to disorder, and indicated that the reaction was a heterogeneous catalytic process occurring on the surface of the catalyst monolayer.

## Abstract 2

“Channel-like” self-assembled monolayers having aliphatic and aromatic diimines (denoted as Si@1DIS, Si@2DIS and Si@3DIS) immobilized on substrates and their palladacycle monolayers (Si@1DIS-Pd, Si@2DIS-Pd and Si@3DIS-Pd) were prepared and characterized.

## Graphical abstract

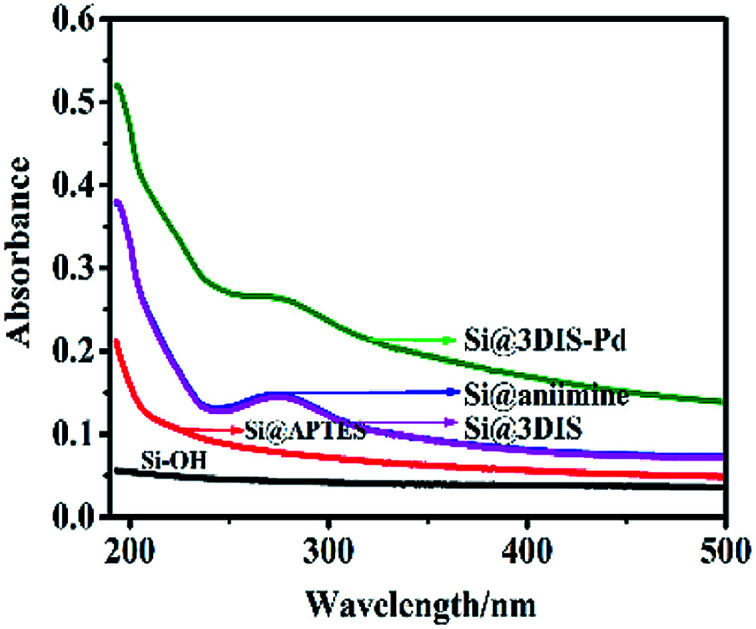

## 1. Introduction

Palladium (Pd)-catalyzed C–C coupling reactions, such as Suzuki, Heck, Sonogashira as well as other reactions, have important roles in organic chemistry^1^ and have made major progress,^2^ including use as homogeneous catalysts. Despite their remarkable usefulness and excellent activity, homogeneous catalysts cannot be recycled, especially in the pharmaceutical industry.^3^ Therefore, heterogeneous catalysts seem to be preferable because they can be removed by simple filtration leaving few metal residues.^4^

Studies on Pd catalysts immobilized on different substrates have been described^5–9^ and applied to catalyze coupling reactions^10^ due to their excellent stability and versatility towards functional groups. However, some problems associated with heterogeneous catalyst must be solved, such as high loading, heavily reaction condition, aggregation on the substrate, and repeatability.^11^ Meanwhile, finding the true mechanism is one of the most striking weaknesses of current studies on heterogeneous catalysis.^12^

Different mechanisms of coupling reactions catalyzed by heterogeneous Pd catalysts have been investigated intensively, including the homogeneous process by leaching Pd species,^13^ the direct role of Pd nanoparticles on surface sites in the catalytic cycle,^14^ and surface-driven coupling.^15^ Despite these efforts, there is uncertain evidence of surface-catalyzed cross-coupling chemistry and some results have been contradictory. Therefore, research should be focused to elucidate the dynamics and true species type of Pd catalyst. Such research may enable development of a rational design for catalysts. Hence, suitable analytical methods must be employed for research.

The surface properties of Langmuir–Blodgett (LB) films^16^ and self-assembled films^17^ immobilized on solid substrates can be characterized, identified and evaluated by various measurement techniques. These techniques provide deep insights into catalytic behaviour at the molecular level, such as in the design of ligands containing certain functional groups (*e.g.*, Schiff-bases), which exhibit excellent performance in heterogeneous catalysis.^18,19^ Our research team focuses on the fabrication and heterogeneous catalytic properties of Schiff-based cyclopalladated LB films^20^ and self-assembly films, which show superior catalytic activity.^21^ LB films can be destroyed in organic solvents, heating or distilling, which affects experimental results. Therefore, their self-assembled monolayers linked with substrates were selected to overcome these disadvantages.^20*b*^

Herein, a series of aliphatic and aromatic cyclopalladated diimine catalysts in the form of “channel like” self-assembled monolayers were designed and prepared to evaluate their activity and recyclability in the Suzuki–Miyaura coupling reaction. In this way, we aimed to provide an “ideal” template for investigating the heterogeneous catalytic mechanism.

## 2. Experimental section

### 2.1 General

Chemical regents were purchased from commercial and experimental sources. The fabrication of cyclopalladated diimines SAM-monolayer (Si@1DIS-Pd, Si@2DIS-Pd and Si@3DIS-Pd), general procedure for the Suzuki cross-coupling reaction as a model, inductively coupled plasma-atomic emission spectroscopy (ICP-AES) analysis and the general procedure for control experiments are presented in ESI.†

## 3. Results and discussion

### 3.1 Preparation of self-assembled monolayers of cyclopalladated diimines (Si@1DIS-Pd, Si@2DIS-Pd and Si@3DIS-Pd)

**Scheme 1:**
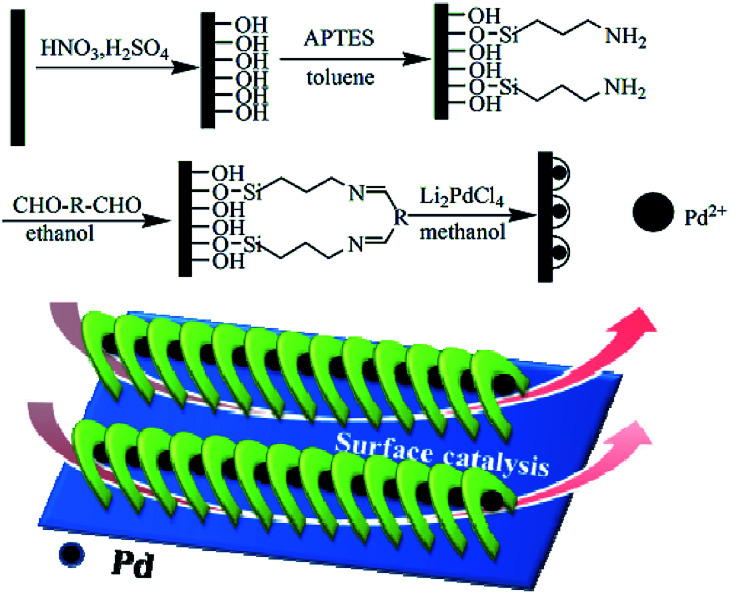
Preparation of cyclopalladated diimine self-assembled monolayers (R = –(CH_2_)_*n*_–: *n* = 0, Si@1DIS-Pd; *n* = 3, Si@2DIS-Pd; R = –C_6_H_4_–, Si@3DIS-Pd).

The channel-like cyclopalladated diimines grafted on substrates, such as silicon, were fabricated as depicted in Scheme 1. The channel diameter could be adjusted by designing the ligands. Characterization of cyclopalladated diimine self-assembled monolayers (Si@1DIS-Pd, Si@2DIS-Pd and Si@3DIS-Pd), including the immobilization process, were carried out and interpreted as shown below.

#### 3.1.1 UV-vis absorption spectra of the preparation process of catalytic monolayers

**Fig. 1:**
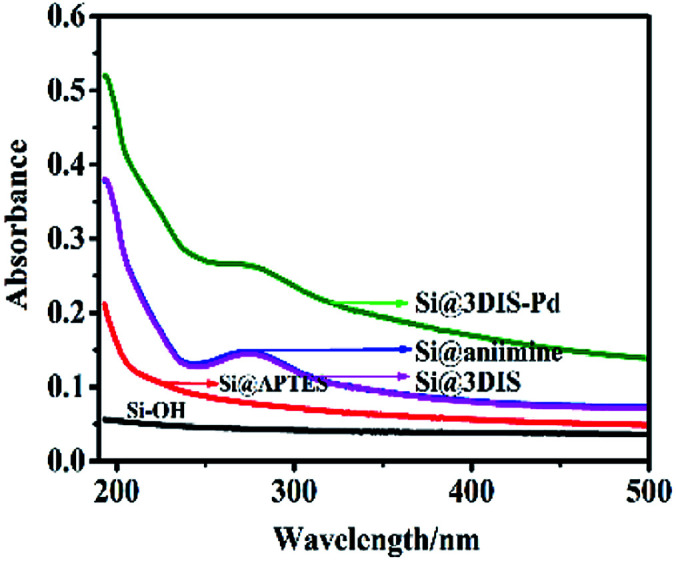
UV spectra of the preparation process of Si@3DIS-Pd at different steps.

UV spectra of the fabrication of monolayers of cyclopalladated diimines (Si@1DIS-Pd, Si@2DIS-Pd and Si@3DIS-Pd) modified on quartz, along with each step, were measured (Fig. 1). For Si@3DIS-Pd, absorption was not observed for hydrophilic quartz (Si–OH, black line) and the absorption increased gradually due to the amine (Si@APTES, red line). Then, a peak at 273 nm assigned to benzene appeared after modification with 1,4-phthalaldehyde (Si@3DIS, pink line). To ascertain if a diimine had been formed, a contrast experiment was initiated in which Si@3DIS was reacted with aniline. Results showed no change (Si@aniimine, blue line) compared with that of Si@3DIS, indicating that an ordered diimine had been fabricated. The peak at 273 nm shifted to 265 nm (green line) when Si@3DIS coordinated with Pd due to decreasing conjugation of the diimine.^20*d*^ The absorption peak shifted to shorter wavelengths gradually, suggesting some interaction among molecules or close molecular packing, such as J-aggregates. Similar results were observed for Si@1DIS-Pd and Si@2DIS-Pd (see ESI, Fig. S1†).

#### 3.1.2 Water contact angle (WCA) analyses

**Fig. 2:**
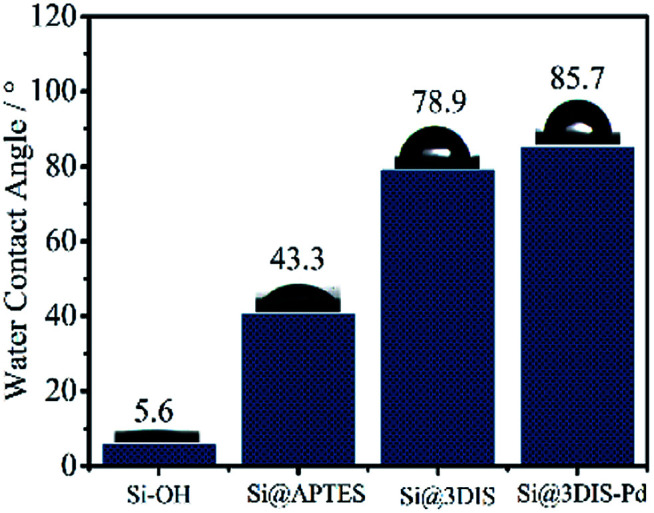
Comparison of the WCA in the preparation of cyclopalladated diimine monolayers (Si–OH, Si@APTES, Si@3DIS, and Si@3SID-Pd).

The WCA is a useful way to observe the surface wetting ability related to the structure of the monolayer.^22^ After pre-treatment with “piranha” solution (Fig. 2), the surface of silicon exhibited higher hydrophilic performance due to hydroxyl groups (5.6°) and the WCA increased to 43.3° due to hydrophobic fatty chains after reaction with APTES. The surfaces linked with 1,4-phthalaldehyde showed a dramatically increased WCA of 78.9° because of introduction of an aryl group. Finally, the WCA of Si@3SID-Pd was 85.7° after coordination with Pd_2_LiCl_4_. Changes in the WCA on modified solid surfaces resulted in significant changes in the surface configuration of each step. In the cases of Si@1DIS-Pd and Si@2DIS-Pd, similar results were shown (Fig. S2†).

#### 3.1.3 Analyses of atomic force microscopy (AFM) images

**Fig. 3:**
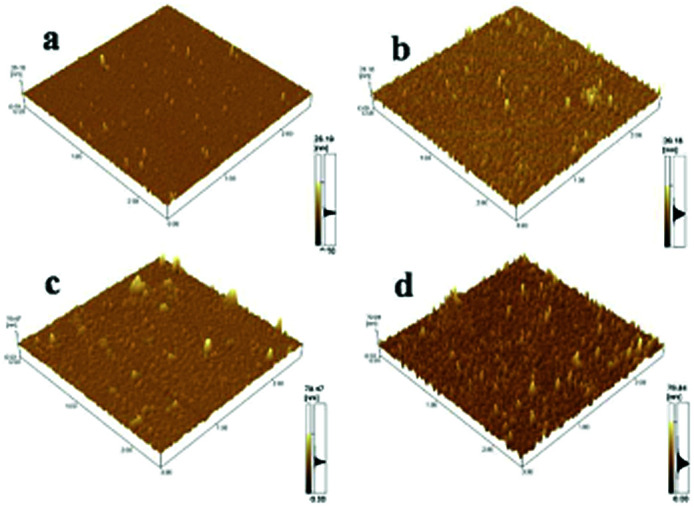
AFM images of Si@3DIS-Pd in different preparation steps ((a) Si–OH, (b) Si@APTES, (c) Si@3SID and (d) Si@3SID-Pd).

AFM images of the surface in derivative processes were measured at different steps to provide detailed information on preparation of Si@1DIS-Pd, Si@2DIS-Pd and Si@3DIS-Pd (Fig. 3). AFM images of hydrophilic silicon (Fig. 3a) showed a flat and ordered morphology with a roughness average (*R*_a_) of 0.540 nm and root mean square (Rms) of 0.797. Ordered surfaces with a *R*_a_ of 1.151 nm and Rms of 1.570 nm (Fig. 3b) were observed after silanization.^23^ The AFM images obtained after addition of 1,4-phthalaldehyde (Fig. 3c) also exhibited homogeneous surfaces except for a few clusters, but a higher *R*_a_ (2.419 nm) and Rms (4.196 nm) than that observed after silanization (1.151 nm and 1.570 nm, respectively), indicating that a diimine could be formed. AFM of the surface of a cyclopalladated diimine monolayer (Fig. 3d) gave high-density arrays with a *R*_a_ of 4.151 nm and Rms of 5.863 nm, suggesting that self-assembly of Pd with Si@3SID had occurred and resulted in changes in morphology. AFM data as well as *R*_a_ and Rms values of different processes for Si@1DIS-Pd and Si@2DIS-Pd are provided as shown in Fig. S3a and b† and summarized in Fig. S4.†

#### 3.1.4 X-ray photoelectron (XPS) spectroscopy

**Fig. 4:**
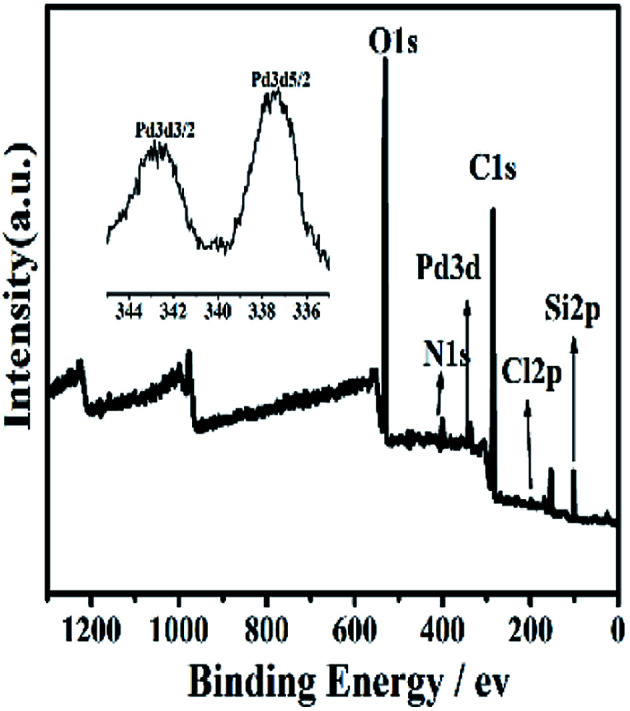
XPS spectra of Si@3SID-Pd. Inset: high-resolution XPS of Pd3d.

The XPS spectra of Si@3SID-Pd (Fig. 4), Si@1DIS-Pd (Fig. S5†) and Si@2DIS-Pd (Fig. S6†) were obtained, which covered the Si2p, Cl2p, C1s, N1s, and Pd3d energy ranges. The binding energies are summarized in Table S1† for Si@1DIS-Pd and Table S2† for Si@2DIS-Pd. Signals appeared in the spectrum of Cl2p at 197.0 eV BE and Pd3d at 343.2 eV BE and 337.9 eV BE, confirming Pd(ii) and chloride on the surface.^21*h*^ These data provided evidence that Si@1DIS-Pd, Si@2DIS-Pd and Si@3DIS-Pd had been grafted on the wafer surface step by step (Table 1).

**Table 1:** Relative element percentage content of Si@3DIS-Pd

Element	Pd3d	Si2p	Cl2p	C1s	N1s	O1s
Atomic%	0.46	8.70	0.60	56.66	5.16	26.85

#### 3.1.5 Cyclic voltammetry (CV)

**Fig. 5:**
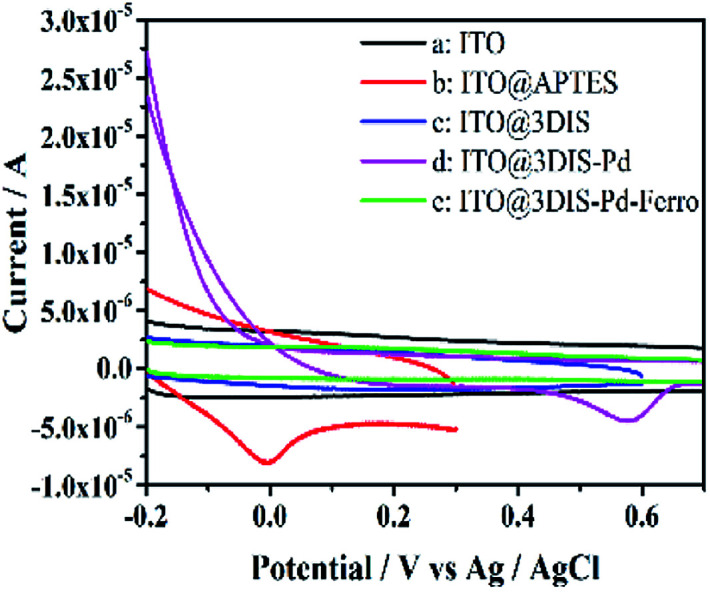
Comparison of cyclic voltammograms of ITO@3DIS-Pd during the preparation. (a) ITO; (b) ITO@APTES; (c) ITO@3DIS; (d) ITO@3DIS-Pd and (e) ITO@3DIS-Pd reacted with 2-ethylamino ferrocene.

The electrochemical properties of cyclopalladated diimines (ITO@3DIS-Pd) at different steps were measured (Fig. 5). An oxidation wave at ∼0 V showed that an amine had appeared after grafting with APTES (Fig. 5b). Voltammograms showed a surface wave consisting of oxidation at ∼0.22 V ascribed to a Schiff-base group when ITO@APTES was grafted with 1,4-phthalaldehyde (Fig. 5c). To ascertain if a diimine (ITO@3SID-Pd) had been formed, a contrast experiment was initiated in which ITO@3DIS-Pd was reacted with 2-aminoethyl-ferrocene. No redox waves of ferrocene appeared,^20^ indicating that a diimine on the surface of the substrate had been prepared (Fig. 5e). An oxidation peak was observed at 0.58 V, which is the characteristic peak of cyclopalladated diimines, suggesting that cyclopalladated diimine was located on the ITO electrode (Fig. 5d). Similar results are presented in Fig. S7 and S8† for ITO@1DIS-Pd and ITO@2DIS-Pd, respectively.

Characterization of the WCA as well as the UV-vis, XPS, AFM and CV data obtained above demonstrated that ordered cyclopalladated diimine self-assembled monolayers were fabricated. Also, the Pd content in cyclopalladated diamine monolayers was 7.7 × 10^−10^, 45.1 × 10^−10^ and 188.67 × 10^−10^ mmol cm^−2^ for Si@1DIS-Pd, Si@2DIS-Pd and Si@3DIS-Pd, respectively. Therefore, they were used to explore surface chemical reactions using the Suzuki coupling reaction as a model.^24^

### 3.2 Catalytic properties and recycling of Si@1DIS-Pd, Si@2DIS-Pd and Si@3DIS-Pd

#### 3.2.1 Investigation of catalytic properties

The Suzuki coupling reaction is usually employed as a template for characterizing catalytic properties.^25^ The best catalytic conditions for use in subsequent catalytic reactions were: 90 (°C), 2–4 h, K_2_CO_3_ as the base, water as the solvent for Si@2DIS-Pd and Si@3DIS-Pd (Table S3†). However, Si@1DIS-Pd showed lower catalytic activity due to the smaller amount of Pd immobilized.

Si@3DIS-Pd had higher activity with less catalyst (Table S4†) compared with that of Si@2DIS-Pd (Table S5†), suggesting that catalytic efficiency is dependent upon the orientation, stability and density of catalysts. Higher yields (97–99%) were obtained in the presence of Si@3DIS-Pd in neat water (Table S4,† entries 1–3 and 5–10), even with electron-rich moieties except for *o*-OCH_3_–C_6_H_4_–Br due to the steric effect (R = *o*-OMe, entry 4; *o*-methyl, entry 11). Especially for 4-chlorobenzaldehyde (Table S4,† entry 12), Si@3DIS-Pd was more catalytically active than that of Si@2DIS-Pd (Table S5,† entry 12) due to the higher stability and ordered orientation of Si@3DIS-Pd. The coupling reactions of derivatives of bromoboronic acids with arylbromides were carried out with Si@3DIS-Pd (Table S4,† entries 14–16) to obtain excellent yields. This difference in catalytic activity was attributed to the easy accessibility of substrates with active sites on monolayer surfaces.

#### 3.2.2 Recyclability and stability of Si@3DIS-Pd

**Fig. 6:**
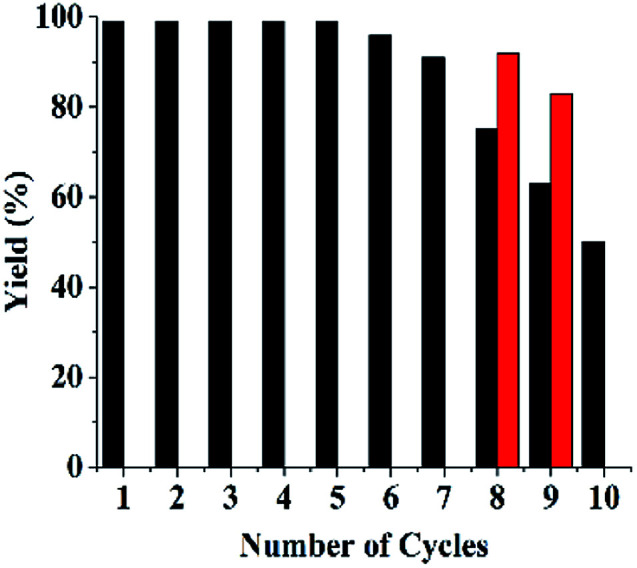
Recycling of Si@3DIS-Pd.

Recycling results showed that the yield decreased after eight cycles, but yields of 4-methylbiphenyl maintained at 50% could be obtained over 10 runs. In the case of Si@1DIS-Pd and Si@2DIS-Pd, they could be reused only twice due to easy hydrolysis of fatty Schiff-base groups. UV changes of Si@2DIS-Pd during a catalytic program (Fig. S9†) showed that the peak at 261 nm of a Schiff-base group decreased with increasing time, resulting in the loss of the catalyst. These results showed that Si@3DIS-Pd with an aryl imine was stable. Also, 90–93% yield could be obtained after several cycles in which the reaction time was lengthened to 10 h after eight cycles (red column) (Fig. 6).^21*h*^

### 3.3 Investigation of the catalytic mechanism

Many efforts have been made to illustrate the changes of active centers and interfaces during catalysis. However, most mechanistic studies have only observed the changes of surfaces and structures before and after the heterogeneous catalytic process.^14*a*^ Detailed research on the whole process needs to be done. It is necessary to carry out leaching studies,^4*b*^ kinetic studies, hot filtration tests and poisoning tests to solve the question of heterogeneity.^26^ Hence, the functional surfaces of monolayer can be designed as “bridges” between homogeneous and heterogeneous systems. Morphologic changes of surfaces during catalysis are important factors for elucidating mechanisms.

#### 3.3.1 Hot filtration tests and dynamic studies

**Fig. 7:**
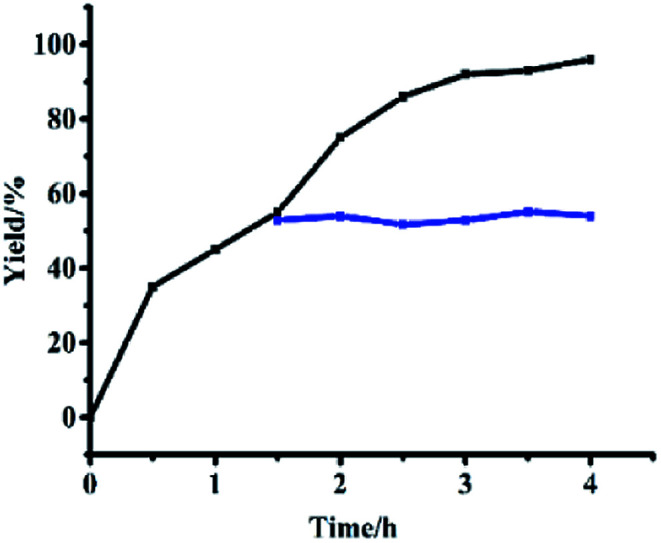
Hot filtration test of Si@3DIS-Pd used for catalysis of the Suzuki coupling reaction.

When a catalyst was added and removed after 1.5 h, 55% yields were obtained (Fig. 7). Yields did not change until 4 h (Fig. 7, blue line). However, when the catalyst was added to the reaction solution, 95% yield could be obtained after 4 h (Fig. 7, black line). These contrast experiments showed that leaching of active catalysts did not occur from the monolayer of functional catalysts.

#### 3.3.2 Catalyst poisoning

The catalytic activity could be inhibited effectively after mercury addition (Table S5†), in which the yield was 28% (Table S6,† entry 1). Mercury cannot make contact sufficiently with the catalytic active site due to its poor dispersibility in the solvent.^21*i*^ In the case of the PPh3 ligand, catalytic activity did not decrease (Table S6,† entry 2), which was attributed to ligand exchange with chlorine. However, when thiophene was presented, a significant decrease in activity was observed because the active sites were covered with thiophene (Table S6,† entry 3). These results showed that the catalytic process occurred mainly on the surface of Si@3DIS-Pd monolayers, which acted as a heterogeneous system.

#### 3.3.3 Investigation of the morphology of catalytic surfaces during catalysis with or without stirring

Stirring is an important factor in catalytic processes (particularly in heterogeneous systems). Stirring can improve the reaction rate because of the equilibrium between absorption and desorption on the surface. Therefore, surface changes as imaged with AFM can be described to understand catalytic mechanisms.

**Fig. 8:**
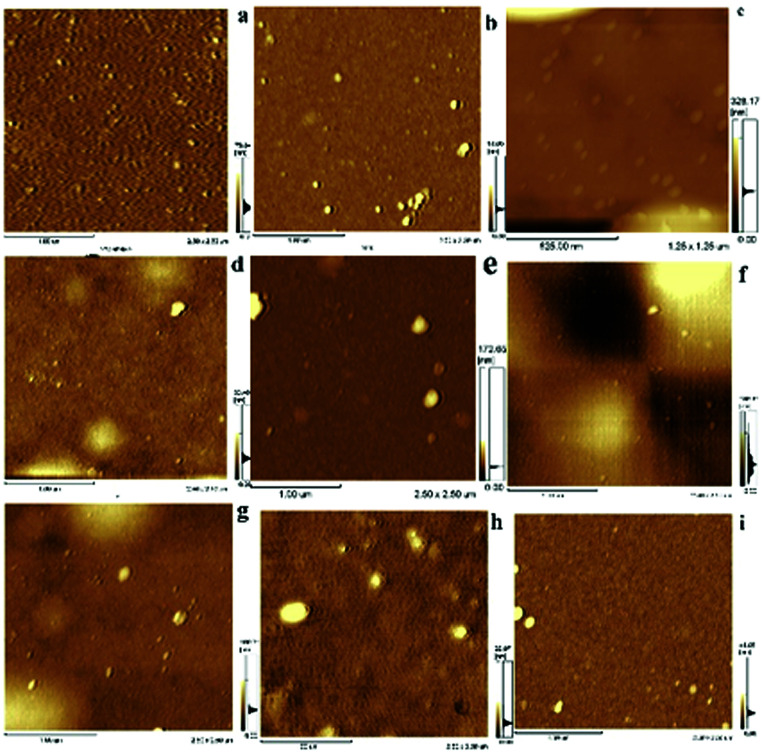
AFM images of Si@3DIS-Pd monolayer during catalytic process with stirring. (a), (b), (c), (d), (e), (f), (g), (h), (i), respectively, represented before and after catalysis at 0.0 h, 0.5 h, 1.0 h, 1.5 h, 2.0 h, 2.5 h, 3.0 h, 4.0 h and ultrasonic treatment after reaction.

To elucidate changes on catalytic surfaces related with the catalytic mechanism, the corresponding AFM images as well as *R*_a_ and Rms values at different reaction time under stirring (Fig. 8, Table 2) or without stirring (Fig. S10 and S11†) were obtained. AMF (Fig. 8) showed some aggregates of height of 4–5 nm before catalysis (Fig. 8a) but the morphology of the catalyst surface changed dramatically with more aggregates of average height 12 nm in 0.5 h (Fig. 8b), indicating that absorption of substrates occurred on the surface of catalyst monolayers. Round aggregates (30 nm) appeared after 1 h (Fig. 8c), which might have been due to automatic agglomeration of coupling products because the absorption rate was higher than the desorption rate. This phenomenon was the key factor for the catalytic process. It can be observed in Fig. 8d and e that the average height was similar to that at 1 h. Significant morphologic changes on catalyst surfaces were observed during 2.5 h of catalytic processes (Fig. 8f), in which the average height reached ∼120 nm and orientation became irregular. This finding suggested that a violent reaction occurred, accompanied by a sequential adsorption and chemical process (including substrates, intermediates and product), on the surface of the catalyst monolayer. The morphology of the catalyst monolayer turned became more regular during 3–4 h compared with that at 2.5 h, with a height range from 20 nm to 40 nm (Fig. 8g and h). Finally, the ordered catalyst surface could be observed after the used catalyst had been treated by ultrasonic stimulation in water (Fig. 8i). The remarkable changes stated above can be illustrated by active palladacycle on the surface of a catalytic monolayer, including absorption, synergism and adsorption.^27^ AFM images and *R*_a_/Rms values at different catalytic reaction times without stirring (Fig. S10 and S11†) were also obtained. Results showed that catalytic time must be increased because the covered substrate or product affects the surface catalytic process due to a slower rate of adsorption. This finding was also evidence that stirring could aid surface catalysis (detailed analysis is presented in ESI†).

**Table 2:** *R*_a_/Rms data from AFM images of Si@3DIS-Pd used for catalysis at different times

Time (h)	0.0	0.5	1.0	1.5	2.0	2.5	3.0	4.0	Ultrasonic
*R*_a_ (nm)	4.15	1.65	16.18	3.14	3.99	25.28	6.69	1.82	1.70
Rms (nm)	5.86	3.56	33.49	4.99	13.38	33.19	10.27	3.46	3.29

#### 3.3.4 WCA analyses

**Fig. 9:**
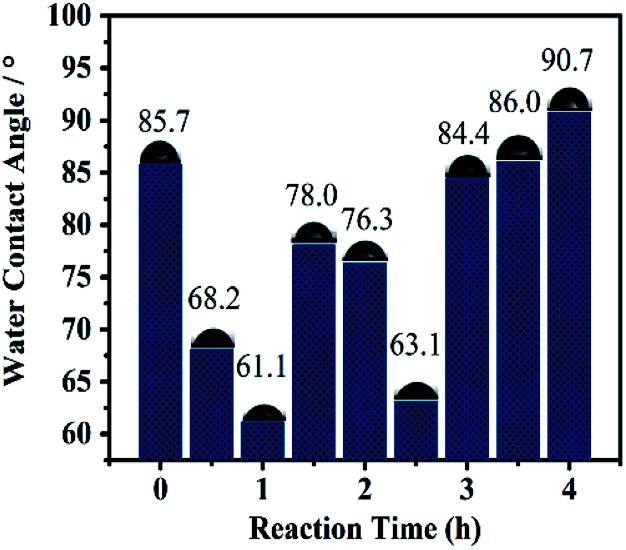
Comparison of static water contact angle of Si@3DIS-Pd during the catalytic process under stirring.

The WCA can be used to observe surface wetting ability.^22^ As catalysis proceeded (Fig. 9), the WCA decreased in 1 h (due to absorption of substrates and salt) and increased before 2.0 h (because of increasing absorption and product). The dramatic decrease in the WAC at 2.5 h was induced by a violent reaction according to AFM (Fig. 8f), and then increased gradually and almost recovered to the original level from 3 h to the end of the reaction, clearly revealing that catalysis proceeded on the surface. A similar phenomenon was presented for catalysis without stirring, in which the catalytic time was prolonged to 24 h (Fig. S12†).

#### 3.3.5 Analyses of cyclic voltammograms

**Fig. 10:**
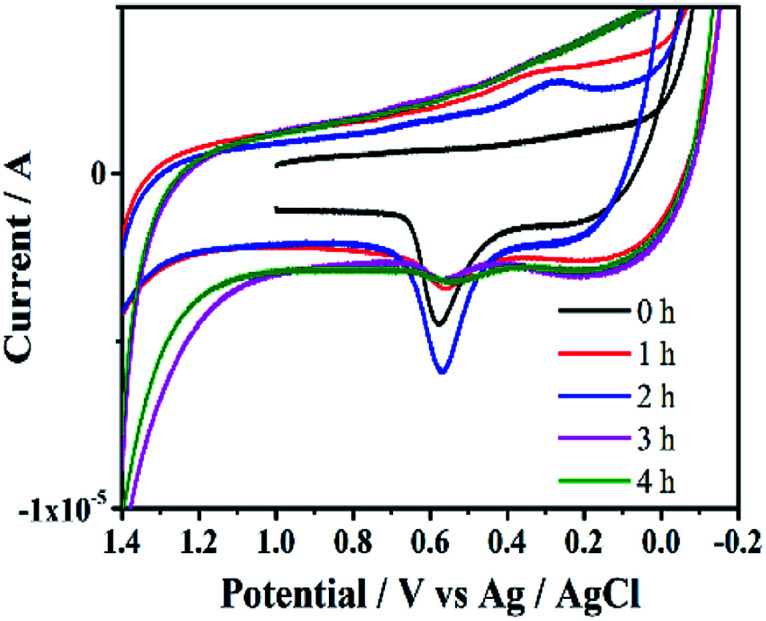
Cyclic voltammograms of Si@3DIS-Pd on ITO during the catalytic process with stirring.

CV can provide important electrochemical information for catalysts at different reaction times, and help to evaluate surface catalytic process. All cyclic voltammograms displayed a pair of weak and broad redox peaks assigned to Pd(0)/Pd(ii) at 0.566 and 0.216 (Fig. 10). The intensity of the peak of Si@3DIS-Pd increased until 2 h, indicating that the catalytic site had lower electroactivity because of redox-active sites covered by some substrates. This finding could be interpreted as the electrode surface being covered gradually by the absorption substrates and intermediates of oxidative addition under catalysis. The peak decreased after 3 h due to adsorption of the coupling product from the catalyst surface. This finding was in agreement with the results of WCA measurement and AFM mentioned above.

#### 3.3.6 XPS investigation of the catalytic surface during catalysis with stirring

**Fig. 11:**
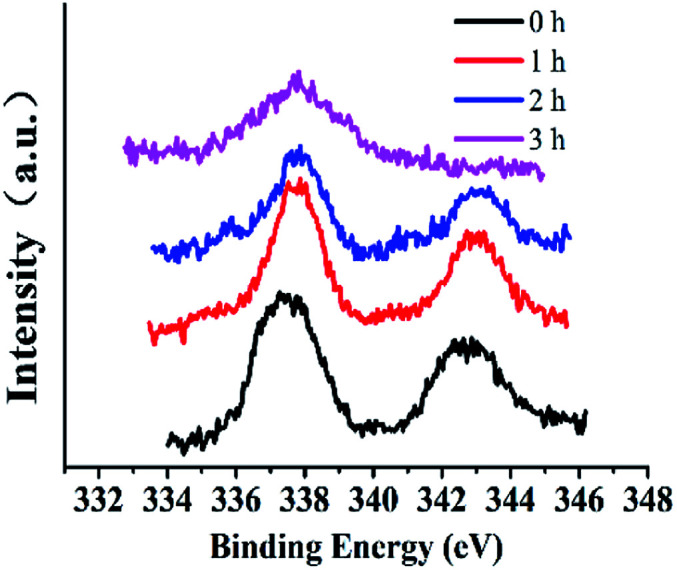
The high-resolution XPS of Pd3d during the catalytic process (Si@3DIS-Pd). (a) 0 h, black line; (b) 1 h, red line; (c) 2 h, blue line; (d) 3 h, pink line.

**Fig. 12:**
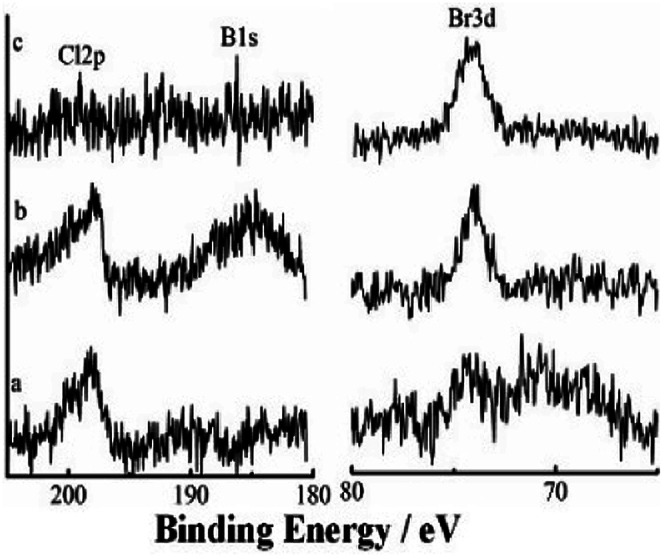
High-resolution XPS of B1s, Cl2p and Br3d during the catalytic process at (a) 1 h, (b) 2 h and (c) 3 h.

XPS spectra can give information that can help understanding catalytic mechanisms.^15^ Changes in active centers were detected through analyses of Si@3DIS-Pd catalysis (Fig. 11). Peaks at 345 eV and 337.8 eV denoted Pd3d 3/2 and Pd3d 5/2 of Pd(ii) before the catalytic reaction (Fig. 11a) was observed. Two new peaks at 340.5 eV and 335.3 eV assigned to Pd3d 3/2 and Pd3d 5/2 of Pd(0) appeared because of the reduction of Pd(ii) during the catalytic process (Fig. 11b) and shifted obviously to lower energy (341.0 eV and 335.8 eV), which was called the “induction period”, indicating generation of new intermediates.^28^ These findings were consistent with the results shown in Fig. 8, during which a violent reaction occurred at 2 h. However, the peaks at 341.0 eV and 335.8 eV disappeared after 3 h and only the peak at 337.7 eV remained, implying that Pd(0) was re-oxidized to Pd(ii) at the end.^29^ When the catalytic process occurred, Br 3d peaks at 68.5 eV and 67.4 eV appeared, indicating that the Pd(0) oxidation with Br–Ph occurred on the surface.^30^ Simultaneously, the B1s peak at 185.5 eV also appeared after 2 h (Fig. 12b), implying that the phenylboronic-acid addition took place. The amount of Pd was 21.7 × 10^−10^ mmol in solution as measured by ICP-AES, showing almost no leaching of Pd. According to the results presented above, the real active center Pd(0) generated *in situ* was stabilized by diimine ligands on the surface.^31^

**Scheme 2:**
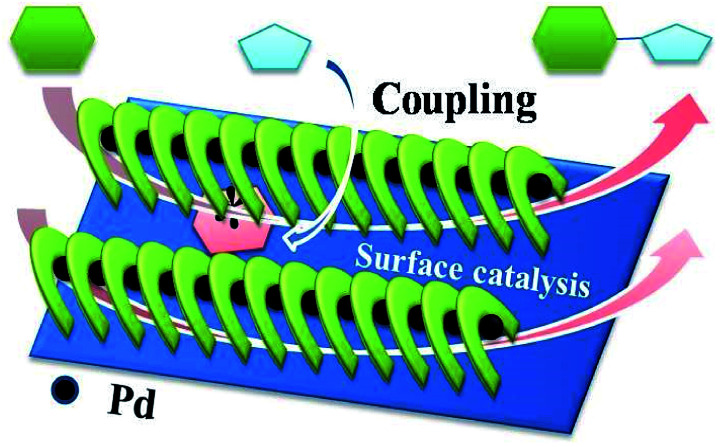
Plausible catalytic process.

Based on the investigations mentioned above, catalytic processes were proposed (Scheme 2). Ph–Br was absorbed and reacted with Pd(0) species on the surface to form oxidation products, followed by insertion with PhB(OH)_2_ to give an organometallic. Finally, reductive elimination of intermediates yielded the target molecules, accompanied by the generation of Pd(0) species to complete the catalytic cycle.^21^

## 4. Conclusions

Channel-like self-assembled monolayers with aliphatic and aromatic diimines (denoted as Si@1DIS, Si@2DIS and Si@3DIS) immobilized on substrates and their palladacycle monolayers (Si@1DIS-Pd, Si@2DIS-Pd and Si@3DIS-Pd) were fabricated and characterized. Their catalytic properties for the Suzuki coupling reaction were investigated. Si@3DIS-Pd showed higher catalytic activity in neat water and better recyclability (at least eight times) without Pd leaching. These features can meet the specifications of the pharmaceutical industry better than that of Si@2DIS-Pd and Si@1DIS-Pd because the carbon atom in the aliphatic diimine of Si@2DIS-Pd and Si@1DIS-Pd was easily hydrolyzed due to an active hydrogen of α-C, resulting in poor recyclability. Control of the amount of catalyst immobilized could be achieved by adjusting the channel-like structure, which also enhanced the catalytic activity. The catalytic process and mechanism were investigated systematically and proposed based on experimental results. AFM showed stirring to be an important factor in the catalytic process (particularly in a heterogeneous system) which could improve the reaction rate because of the equilibrium between sorption and desorption on the surface. Results indicated that catalysis was heterogeneous on the surface of the monolayer. These results will allow a rational method to enhance the activity, selectivity and application of heterogeneous catalysts.

## Conflicts of interest

There are no conflicts.

## Supplementary Material

RA-008-C8RA06365F-s001
