# Supplementary material for: Fabrication and catalytic properties of ordered cyclopalladated diimine monolayer : investigation on catalytic mechanism
Source: RSC Adv. 2018 Sep 12;8(56):31860–7. doi: 10.1039/c8ra06365f (PMC9085729; doi:10.1039/c8ra06365f)
Supplement: RA-008-C8RA06365F-s001 [file RA-008-C8RA06365F-s001.pdf]

Electronic Supplementary Material (ESI) for RSC Advances.
This journal is © The Royal Society of Chemistry 2018

Supporting Information

Fabrication and Catalytic Properties of Cyclopalladated Diimine Self-Assembly Monolayer for Catalyzing Coupling Reaction

Linhong Wang,[1] Pingping Huang,[1] Jun Yang[2], Tiesheng Li[1*], Luyuan Mao[2], Minghua Liu[3*], Yangjie Wu[1*]

[1]*College of Chemistry and Molecular Engineering, The Key Lab of Chemical Biology and Organic Chemistry of Henan Province, The Key Lab of Nano-information Materials of Zhengzhou, Zhengzhou,450001, P. R. China. E-mail: lts34@zzu.edu.cn. Fax :(+)86-371-67766667*

[2]*College of Materials Science and Engineering, Zhengzhou University, Zhengzhou, 450001, P. R. China.*

[3]*Beijing National Laboratory for Molecular Science, Institute of Chemistry, Chinese Academy of Sciences, Beijing 100190, P.R China.*

1. Experimental Section

1.1. General

All solvents were obtained from commercial sources and dried and freshly distilled prior to use. All other chemicals were used as purchased. The catalyst films were performed on commercially available silicon, quartz slides (usually cut into 10 mm×10 mm or 30 mm×10 mm) which were hydrophilic treatment by piranha solution (H_2SO_4/H_2O_2 (7:3; v: v)) for 3.0 h at 90 °C to remove any organic residues and to create silanol groups on the surfaces. UV–vis spectra were recorded with a Lambda 35 UV–vis spectrophotometer (Perkin Elmer Inc. USA). X-ray photoelectron spectroscopy (XPS) data were obtained with an ESCALab220i-XL electron spectrometer from VG Scientific using 300W AlK_α radiation. The base pressure was about 3×10^{-9} mbar. The binding energies were referenced to the C1s line at 284.8 eV from adventitious carbon. A SPM-9500 J3 (Shimadzu Corporation, Japan) was employed for AFM measurements in air at ambient. Cyclic Voltammetry (CV) curves were obtained by CH 1650A, ITO glasses electrode (30×10×1 mm) was used as working electrode. The reference electrode was a saturated calomel electrode (SCE), while the counter electrode was a Pt wire. The electrolytic medium was H_2O (Millipore Q-grade, 18.2 MU cm) containing 0.1 M HCl. All the Suzuki reaction was

accomplished without the protection of inert gas. HPLC was conducted on a Waters 600 liquid chromatography. The HPLC conditions were a Zorbax eclipse Xdb C18 column (150 4.6 mm, 5 lm) with, a flow rate1 mL/mim. The HPLC conditions were a Kromasil C18 column (150×4.6 mm, 5 mm). Inductively coupled plasma atomic emission spectroscopy (ICP-AES) was carried out with ICAP 6000 Series, Thermo Scientific.

1.2 Fabrication of cyclopalladated diimines SAM-monolayer (**Si@1DIS-Pd, Si@2DIS-Pd and Si@3DIS-Pd)**

The freshly hydroxyl silicon or quartz surfaces were placed in a solution of 0.5 mL of 3-aminopropyl triethoxysilane (2.1 mmol) in 10 mL of ethanol. After being treated at 80 °C for 24 h, and then washed successively with toluene, methanol, water, and acetone, dried in a nitrogen stream. The amino-functionalized substrates were immersed in 10 mL of dried ethanol, containing dialdhyde at 70 °C for 7 h to give rise to the "bridge like" surfaces. then resulting substrates were rinsed with copious amounts of ethanol, deionized water, acetone, and subsequently introduced into 10 mL dried methanol containing Li_2PdCl_4 for 48 h at room temperature to produce the cyclopalladated diimines catalyst self-assembly monolayer (SAM). After the reaction, the catalyst-grafted substrates were washed thoroughly with methanol, and acetone, prior to being dried in a nitrogen stream.

1.3. General procedure for the Suzuki cross-coupling reaction of aryl halide with phenyl boronic acid

Aryl halide (0.125 mmol), phenylboronic acid (0.15 mmol), K_2CO_3 (0.15 mmol) and *n*-Bu_4NBr (0.15 mmol) were combined with 3 mL water in a small round-bottom flask used for ‘‘homogeneous’’ and ‘‘heterogeneous’’ runs. The reaction mixture was stirred at 80 °C for 4 h, then, the mixture was acidified, and the product was extracted by ethyl ether for three times. The combined organic phase was dried with $MgSO_4$, filtered, and solvent was removed on a rotary evaporator. Crude product was transferred into a 10 mL volumetric flask to fix its quantity in ethyl acetate. The yields were determined by high performance liquid chromatography (HPLC), based on the peak area ratio between bromobenzene and the product. The HPLC conditions were a

Kromasil C18 column (150×4.6 mm, 5 mm) with methanol/acetonitrile as the mobile phase, a flow rate 0.8 mL/min, column temperature 25 °C, UV-detection wavelength at 272 nm (phenylboronic acid).

1.4 ICP-AES analysis

The amounts of cyclopalladated ferrocenylimines immobilized on solid substrates were measured by inductively coupled plasma atomic emission spectroscopy (ICP-AES) (ICAP 6000 Series, Thermo Scientific). The sample treatments were as follows: **Si-Cat.** (quartz plate or round-bottomed flask) was boiled in concentrated nitric acid, and then, residual solid was dissolved with 2 M hydrochloric acid and transferred into a 10 mL volumetric flask to fix its quantity in water.

1.5 General procedure for controlled trials

First, one of **Si@1DIS-Pd, Si@2DIS-Pd** and **Si@3DIS-Pd** substrates was put into round-bottom flask with aryl halide (0.125 mmol), phenylboronic acid (0.15 mmol), K_2CO_3 (0.15 mmol), *n*-Bu_4NBr (0.15 mmol) and 3 mL water. Then it was taken out from the reaction mixture after three hours, yields of product was detected. No more products could be obtained if the reaction continued to 12 hours without **Si@3DIS-Pd**. However, when the substrate (**Si@-3DIS-Pd**) was put back into a new reaction solution, a yield of products could be obtained after 4 hours again.

2. Characterization of cyclopalladated diimine monolayer

2.1 UV–vis absorption spectra

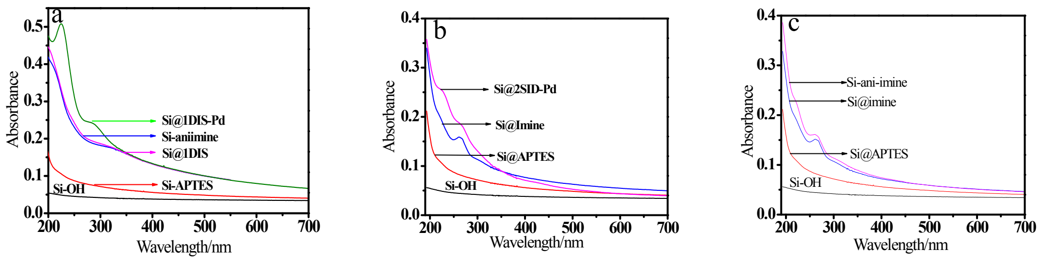

Figure S1 UV–vis absorption spectra of cyclopalldated diimine films. a: **Si@1DIS**, b: **Si@2DIS**, c: **Si@2DIS** reacted with aniline (**Si-aniimine**).

2.2 Water contact angle

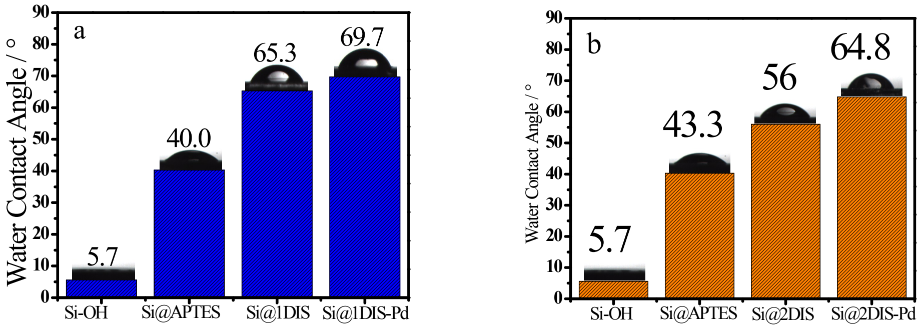

Figure S2. Water contact angles in the progress of preparing cyclopalldated diimine monolayers. (a: **Si@1DIS-Pd**; b: **Si@2SID-Pd**)

2.3 AFM images

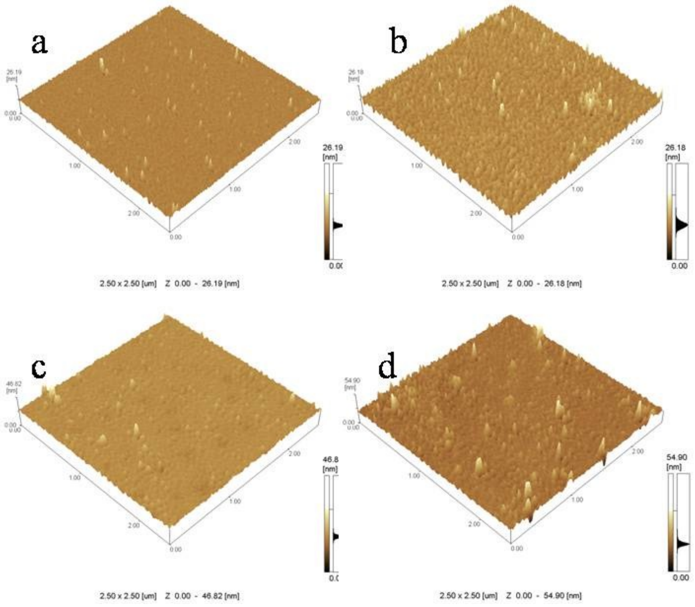

Figure S3a. AFM images of during the preparation process. (a, b, c and d represent **Si-OH**, **Si@APTES**, **Si@1DIS** and **Si@1DIS-Pd**, respectively.)

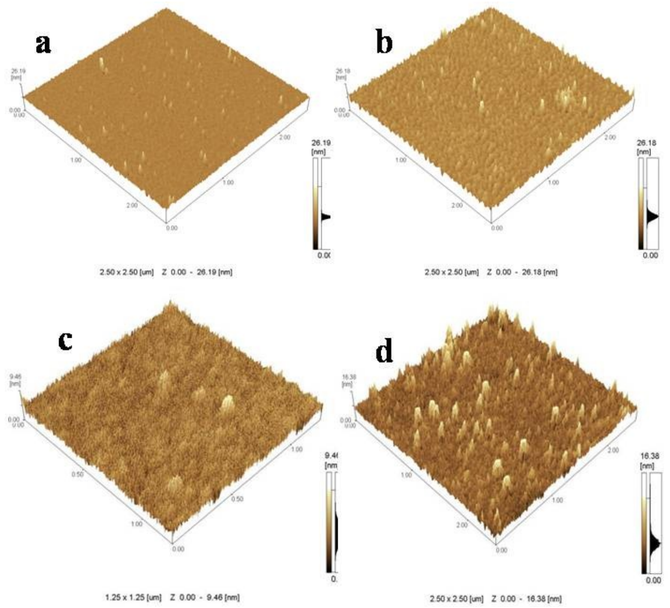

Figure S3b. AFM images of during the preparation process. (a, b, c and d represent **Si-OH**, **Si@APTES**, **Si@2DIS** and **Si@2DIS-Pd**, respectively.)

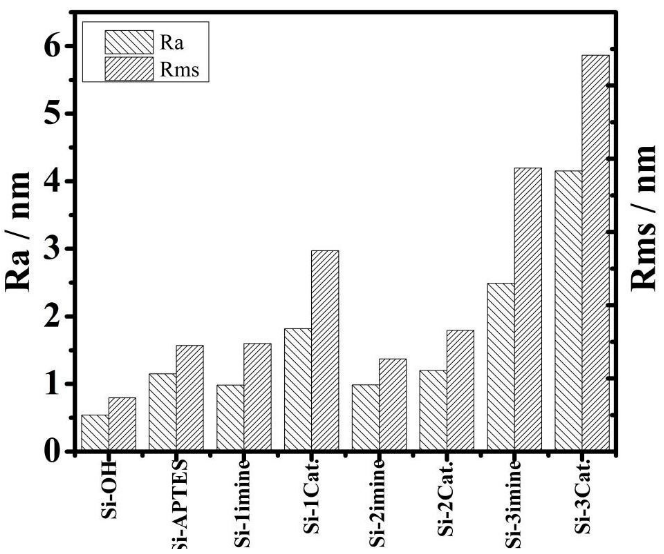

Figure S4. Comparsion of the Ra and Rms of cyclopalldated diimine monolayers during the preparation process. **Si-OH**, **Si@APTES**, **Si-imine**, **Si-Cat**. (**Si@1DIS-Pd** and **Si@2DIS-Pd**), respectively.

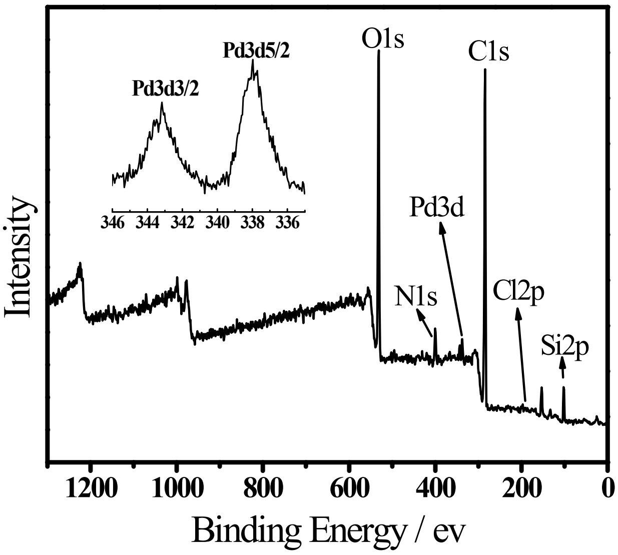

Figure S5. XPS survey spectra of **Si@1DIS-Pd**. Inset: high-resolution XPS of Pd3d.

Table S1 The relative element percentage content of **Si@1DIS-Pd**.

Element	Pd3d	Si2p	Cl2p	C1s	N1s	O1s
Atomic %	0.35	5.9	0.42	64.78	4.44	23.11

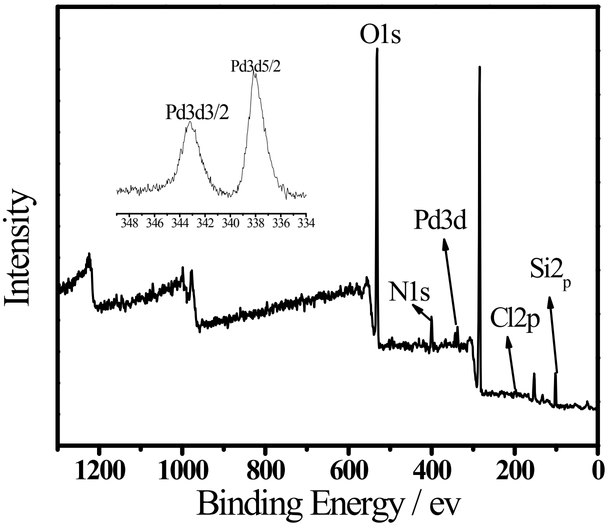

Figure S6. XPS survey spectra of **Si@2DIS –Pd**. Inset: high-resolution XPS of Pd3d.

Table S2 The relative element percentage content of **Si@2DIS-Pd**.

Element	Pd3d	Si2p	Cl2p	C1s	N1s	O1s
Atomic %	0.90	10.88	1.84	60.08	5.66	20.64

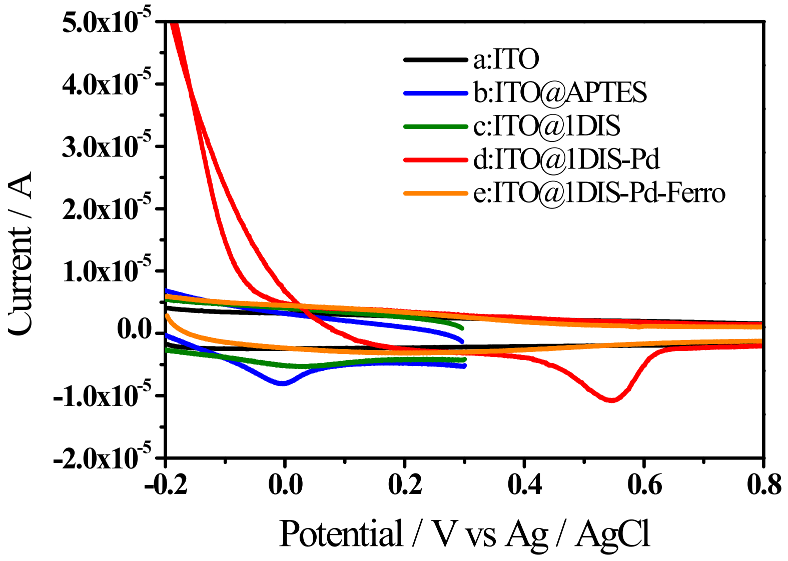

Figure S7. The electrochemical measurements of **Si@1DIS-Pd** monolayer prepared at different steps. a) ITO; b) ITO@APTES; c) ITO@3DIS; d) **ITO@1DIS-Pd** e) **ITO@1DIS-Pd** reacted with 2-ethylamino ferrocene.

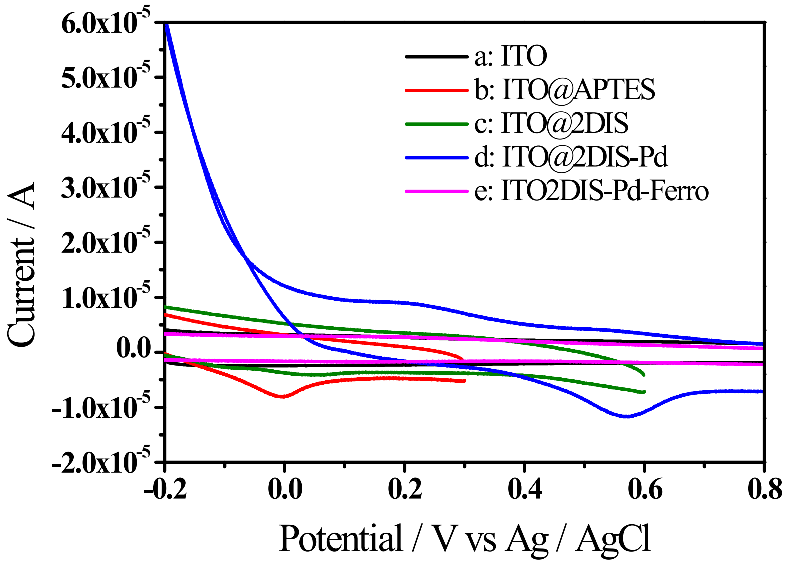

Figure S8. The electrochemical measurements of **Si@2DIS-Pd** prepared at different steps. a) ITO; b) ITO@APTES; c) ITO@3DIS; d) **ITO@2DIS-Pd** e) **ITO@2DIS-Pd** reacted with 2-ethylamino ferrocene.

Table S3 Optimization of temperature and reaction time

Catalyst	*Entry*	*T* (℃)	*t* (h)	Base	Yield (%)[b]	TON ($\times10^4$)
**Si@1DIS-Pd**	1	60	4	K_2CO_3	5	0.156
**Si@1DIS-Pd**	2	80	24	K_2CO_3	21	0.638
**Si@1DIS-Pd**	3	80	4	K_2CO_3	11	0.357

**Si@1DIS-Pd**	4	80	24	K_2CO_3	16	0.525
**Si@1DIS-Pd**	5	90	4	K_2CO_3	20	0.653
**Si@2DIS-Pd**	6	40	4	K_2CO_3	29	0.644
**Si@2DIS-Pd**	7	60	4	K_2CO_3	68	1.513
**Si@2DIS-Pd**	8	80	4	K_2CO_3	95	2.112
**Si@2DIS-Pd**	9	90	4	K_2CO_3	97	2.144
**Si@2DIS-Pd**	10	90	1	K_2CO_3	38	0.844
**Si@2DIS-Pd**	11	90	2	K_2CO_3	43	0.963
**Si@2DIS-Pd**	12	90	3	K_2CO_3	91	2.023
**Si@2DIS-Pd**	13	90	5	K_2CO_3	98	2.184
**Si@3DIS-Pd**	14	40	4	K_2CO_3	33	0.175
**Si@3DIS-Pd**	15	60	4	K_2CO_3	54	0.286
**Si@3DIS-Pd**	16	80	4	K_2CO_3	97	0.514
**Si@3DIS-Pd**	17	90[a]	4	K_2CO_3	82	0.870
**Si@3DIS-Pd**	18	90	2	K_2CO_3	95	0.503
**Si@3DIS-Pd**	19	90	4	K_2CO_3	99	0.525
**Si@3DIS-Pd**	20	90	6	K_2CO_3	99	0.525
**Si@3DIS-Pd**	21	80	4	K_2CO_3	0	
**Si@3DIS-Pd**	22	80	0.5	K_2CO_3	35	0.464

[a]double substrates.

[b]isolated yield

Table S4 Suzuki coupling of aryl Iodides, bromides and chlorines with phenyl boronic acid[a].

(Si@3DIS-Pd)

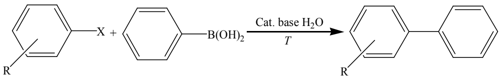

Entry	R-Ar-X	Product	Yield（%）[b]
1	*o*-CHO-C_6H_4-Br	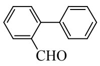	99
2	*m*-CHO-C_6H_4-Br	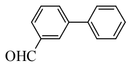	99
3	*p*-CHO-C_6H_4-Br	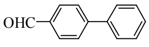	99
4	*o*-OCH_3-C_6H_4-Br	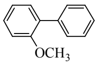	11
5	*m*-OCH_3-C_6H_4-Br	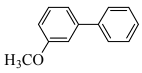	99
6	*o*-NO_2-C_6H_4-Br	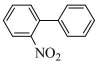	99
7	*m*-NO_2-C_6H_4-Br	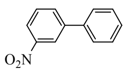	99
8	*p*-NO_2-C_6H_4-Br	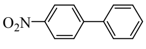	97
9	*o*-CN-C_6H_4-Br	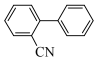	95
10	*p*-CN-C_6H_4-Br	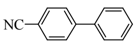	99

11	*o*-CH_3-C_6H_4-Br	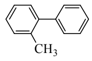	65
12	*p*-CHO-C_6H_4-Cl	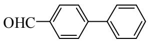	35
13	*p*-CHO-C_6H_4-I	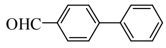	95
14	*m*-CH_3-C_6H_4-$B(OH)_2$	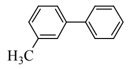	97
15	*p*-OCH_3-C_6H_4-$B(OH)_2$	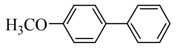	99
16	*o*-C_4H_3S-C_6H_4-$B(OH)_2$	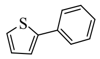	99

[a]Reaction condition: 4-bromotoluene 0.25 mmol, $PhB(OH)_2$ 0.3 mmol, K_2CO_3 0.30 mmol, TBAB(n-Bu_4NBr) 0.30mmol, H_2O 3 mL, 90 ºC, Quartz plate (30 mm×10 mm×1 mm); b) yields determined by HPLC, based on the products.

Table S5 Suzuki coupling of aryl Iodides, bromides and chlorines with phenyl boronic[a] acid (**Si@2DIS-Pd**).

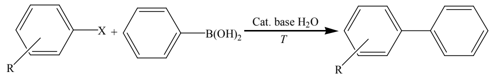

Entry	R-Ar-X	Product	Yield (%)[a]
1	*o*-CHO-C_6H_4-Br	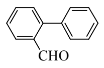	97
2	*m*-CHO-C_6H_4-Br	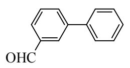	99
3	*p*-CHO-C_6H_4-Br	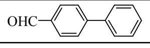	99
4	*o*-OCH_3-C_6H_4-Br	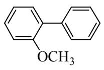	trace
5	*m*-OCH_3-C_6H_4-Br	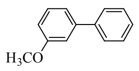	76
6	*o*-NO_2-C_6H_4-Br	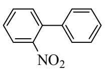	99
7	*m*-NO_2-C_6H_4-Br	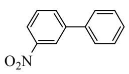	99
8	*p*-NO_2-C_6H_4-Br	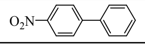	99
9	*o*-CN-C_6H_4-Br	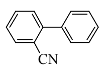	72
10	*p*-CN-C_6H_4-Br	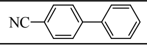	99
11	*o*-CH_3-C_6H_4-Br	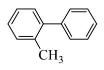	43
12[b]	*p*-CHO-C_6H_4-Cl	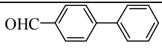	NR
13	*p*-CHO-C_6H_4-I	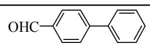	99

a: HPLC, base on the product; b: 12h

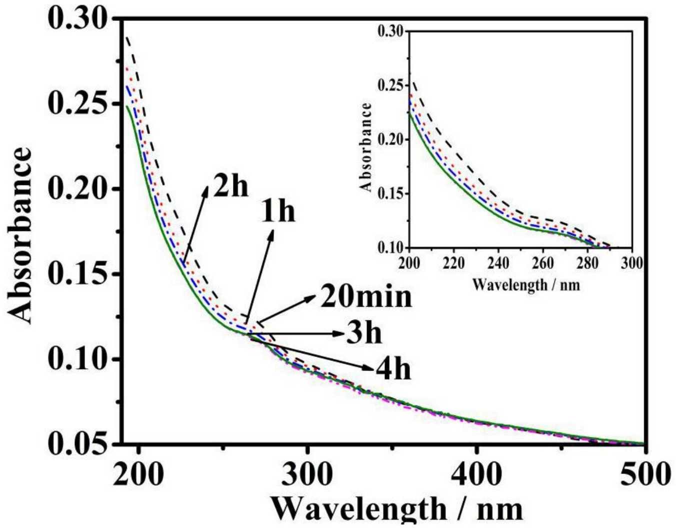

Figure S9. The UV-visible absorption spectrum of **Si@2DIS-Pd** in the catalytic process.

Table S6. Poisoning experiments of **Si@3DIS-Pd**

Entry	Poisoning additive	Isolated yield (%)
**1**	Hg	28
**2**	PPh3	98
**3**	thiophene	9

[a] Reaction condition: benzyl bromide(0.125mmol), phenylacetylene (0.15mmol), sodium azide (0.15mmol), sodium ascorbate(5mg), solvent (7 mL), under N2 atmosphere, RT,16h, substrate: 3cm*0.6 cm*0.8 cm.

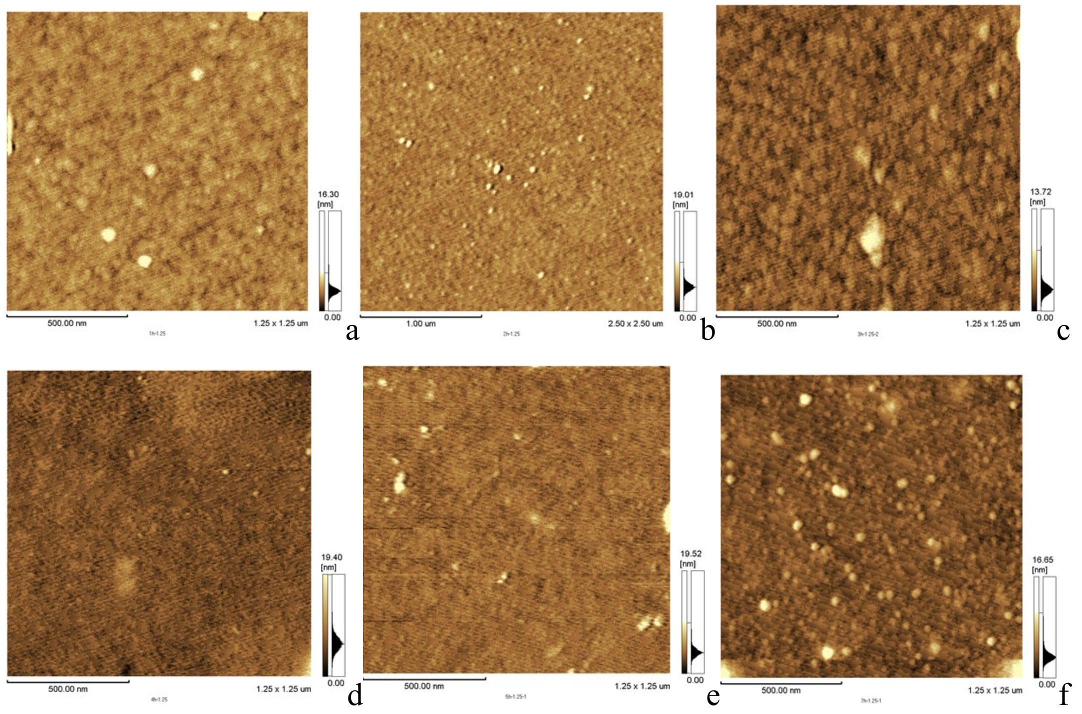

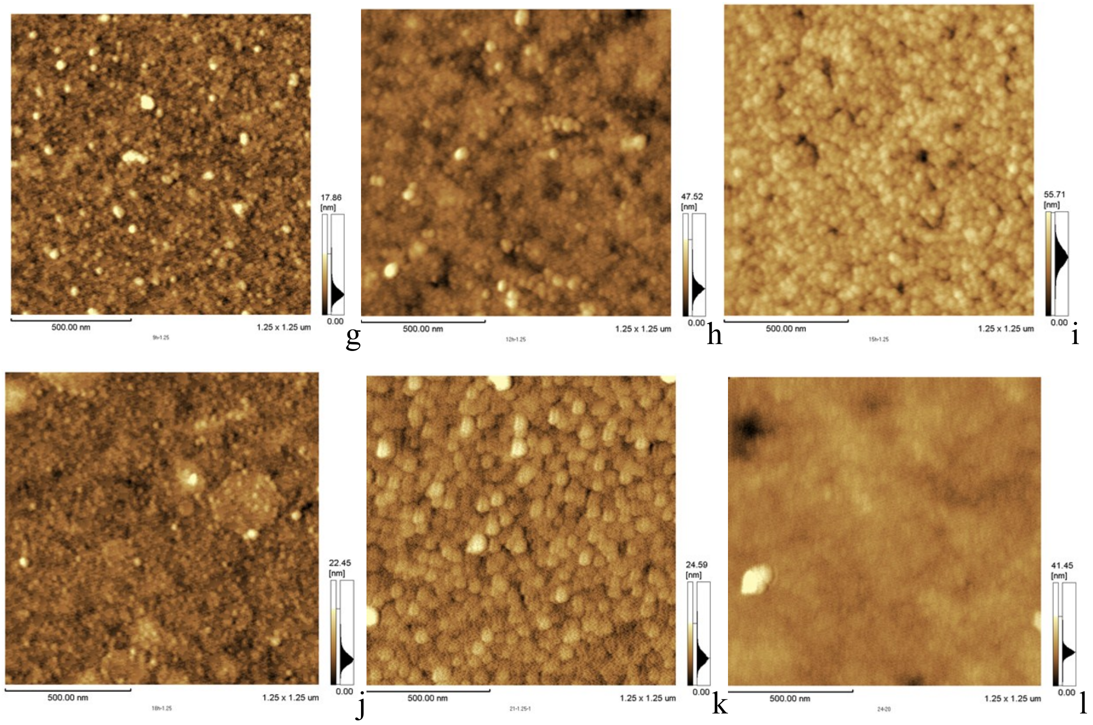

Figure S10. The AFM particle size analysis chart of **Si@3DIS-Pd** during the catalytic process without stirring, a, b, c, d, e, f, g, h, i, j, k, l, respectively. Represent: a) 1 h, b) 2 h, c) 3 h, d) 4 h, e) 5 h, f) 7 h, g) 9 h, h) 12 h, i) 15 h, j) 18 h, k) 21 h, l) 24 h.

Granulometer analysis as shown in Figure S10 was discussed as follow: The surface Ra/rms of AFM images increased and decreased with the catalysis process, the water contact angle increased before 15 h and decreased after 15 h, then gradually increased from 3 h to the end, which vividly revealed that the reaction processes that occurred on the catalyst surface (Figure S12). There were almost no aggregates on the ordered catalyst surface with average height of 3.9 nm between 1h and 5 h were observed (Figure S10a-e), indicating that absorption of substrates was main step. The morphology of catalyst surface changed while more aggregates of 6.6 nm in 7 h-9h appeared (Figure S10f) and average height increased from 12 nm to 19 nm, suggesting that coupling product were formed on the surface of catalyst monolayer. It was noted that larger recognizable coral aggregates were observed in 12-15 h (Figure 10i-j) and the average height increased from 25 to 38 nm, which might be due to the automatically agglomerated of coupling product because of violent reaction rate while absorption and adsorption exist at the same. Then, cluster containing flake and some aggregates could be observed and the average height was 11 nm after 18 h. This agglomerated phenomenon affected the whole catalytic process which could be observed in next steps shown in Figure 10k, in which spherical aggregates with close

accumulation formed by arrangement of aggregates, in which the average height was similar with that of 1 h. The morphology of the catalyst films turned into more regular after 24 h compared with that of 21 h with height range from 20 to 40 nm (Figure S10l). Remarkable changes above could be illustrated by the surface catalysis made by the catalytically active palladcycle on catalyst films including absorption, synergism and adsorption, but reaction time must be prolonged due to no stirring which could efficient affect the adsorption and adsorption during catalytic process. Significant morphological changes of catalyst surface (Figure S10) meant that the reaction occurred on the surface accompanying a sequential adsorption and chemical process occurred on the surface of catalytic monolayer.

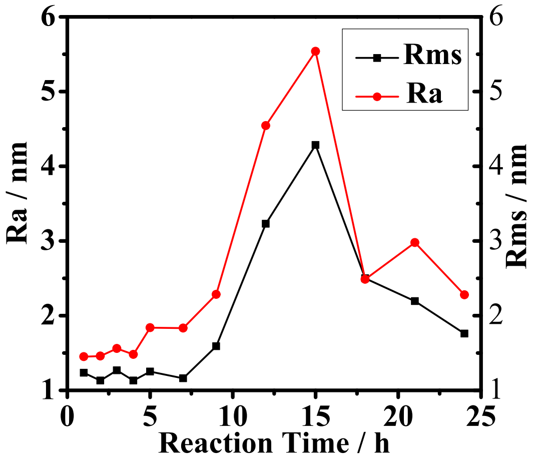

Figure S11. The comparison of Ra and Rms of **Si@3DIS-Pd** during the catalytic process without stirring.

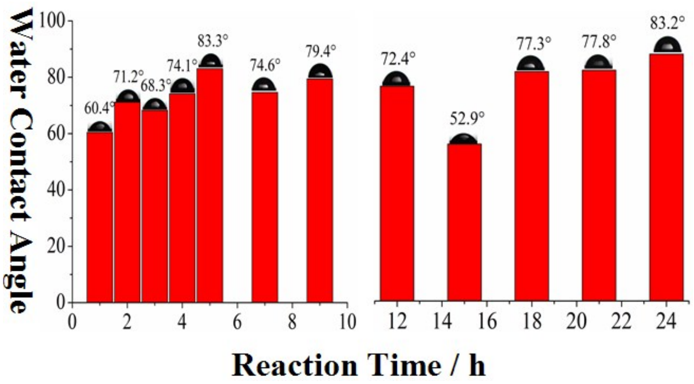

Figure S12. The comparison of static water contact angle of **Si@3DIS-Pd** during the catalytic process without stirring.
